# CircRNA-0100 positively regulates the differentiation of cashmere goat SHF-SCs into hair follicle lineage via sequestering miR-153-3p to heighten the KLF5 expression

**DOI:** 10.5194/aab-65-55-2022

**Published:** 2022-02-22

**Authors:** Junyin Zhao, Jincheng Shen, Zeying Wang, Man Bai, Yixing Fan, Yubo Zhu, Wenlin Bai

**Affiliations:** College of Animal Science & Veterinary Medicine, Shenyang Agricultural University, Shenyang, 110866, P.R. China

## Abstract

Circular RNAs (circRNAs) have stable structures, being a covalently closed loop without 5 ${}^{\prime }$ and 3 ${}^{\prime }$ free ends. They can function as “miRNA sponges” in regulating the expression of their target genes. It was thought that circRNAs are involved in the development of the secondary hair follicle (SHF) in cashmere goats. In our previous investigation, a new circRNA named circRNA-0100 was identified from the SHF of cashmere goats, but its function is unknown. In this work, we found that circRNA-0100 exhibited significantly higher expression at anagen SHF bulge than its counterpart at telogen in cashmere goats. Based on the use of both overexpression and siRNA interference assays, our data indicated that circRNA-0100 promoted the differentiation of cashmere goat SHF stem cells (SHF-SCs) into hair follicle lineage, which was evaluated by analyzing the transcriptional level changes of six indicator genes in SHF-SCs of cashmere goats. Using the RNA pull-down technique, we showed that circRNA-0100 served as “molecular sponges” of miR-153-3p in SHF-SCs. Through the use of dual-luciferase reporter assays, our data indicated that circRNA-0100 positively regulated the transcriptional expression of the KLF5 gene via the miR-153-3p-mediated pathway. Ultimately, we showed that circRNA-0100 promoted the differentiation of SHF-SCs into hair lineage, which might be achieved via sequestering miR-153-3p to heighten the KLF5 expression in SHF-SCs of cashmere goats. Our results provide novel scientific evidence for revealing the potential molecular regulatory mechanisms on the differentiation of SHF-SCs into hair lineage in cashmere goats.

## 1 Introduction

Cashmere, a precious natural protein fiber, is a high-end textile material. The products made from cashmere have been favored by consumers due to its unique lightness, softness, smoothness, delicate feel, and good warmth retention (Jiao et al., 2020). Cashmere is from the secondary hair follicles (SHFs) of skin tissue in cashmere goats. As one of the main products of cashmere goats, cashmere occupies a very important position in the economic income of local farmers and herdsmen in cashmere goat distribution areas (Zhang et al., 2014). The biological events of the differentiation of secondary hair follicle stem cells (SHF-SCs) into hair lineage are essential for the regeneration of SHF and the formation and growth of cashmere fiber in cashmere goats. The SHF-SCs are mainly found in the bulge of SHFs, and during telogen, they are under a status with a slow cycle (Wang and Yin, 2014). However, in the onset phase of SHF anagen, SHF-SCs are induced to activate and proliferate under the signaling derived from dermal papilla cells (DPCs). Further, the SHF-SCs are induced to differentiate into hair lineages in order to promote the SHF regeneration with subsequent formation and growth of cashmere fibers in cashmere goats (Jaks et al., 2010; Morgan, 2014).

In previous investigations, it was demonstrated that the differentiation of hair follicle stem cells into hair lineages was jointly regulated by a variety of endogenous regulatory factors (Lien et al., 2014). There is evidence that overexpression of β -catenin can induce hair follicle stem cells to differentiate into hair lineages to form hair follicles, while the mutation and/or knockdown of β -catenin gene can block the differentiation of hair follicle stem cells into hair lineages, thereby promoting hair follicle stem cells to differentiate into epidermal cells (Zhang et al., 2013a; Choi et al., 2013). There are also other molecules implicated in the differentiation events of hair follicle stem cells into hair lineages, like Lef1 (Zhang et al., 2013b), TCF3 (Amelio et al., 2013; Lien et al., 2014), c-myc, Jagged1, Lhx2 (Folgueras et al., 2013; Shen et al., 2017), and Foxi3 (Shirokova et al., 2016). In recent investigations, it was recorded that some non-coding RNAs were also implicated in the differentiation process of hair follicle stem cells into hair lineages. For example, miR-125b was found to negatively regulate the differentiation of hair follicle stem cells into hair lineages (Zhou et al., 2017), while lncRNA-PlncRNA 1 was demonstrated to promote the differentiation of hair follicle stem cells into hair lineages through upregulating the Wnt / β -catenin signals (Si et al., 2018).

Circular RNAs (circRNAs), as a novel kind of RNA, have stable structures, being a covalently closed loop without 5 ${}^{\prime }$ and 3 ${}^{\prime }$ free ends (Li et al., 2019). CircRNA-producing host genes tend to be longer than average with more exons; moreover, reverse-splicing acceptor exons are highly enriched at the ordinal position 2 of the host genes (Ragan et al., 2019). CircRNAs were progressively identified in various species, and they are thought to be deeply implicated in the gene expression through post-transcriptional regulation (Petkovic et al., 2021). Increasing lines of evidence indicate that many circRNAs play important biological roles by serving as miRNA “sponges” to regulate protein function or by being translated into polypeptides with a physiological function (Hansen et al., 2013; Guo et al., 2014; Kristensen et al., 2019). In humans, it was demonstrated that many circRNAs were involved in various diseases including cardiovascular diseases, neurological disorders, diabetes mellitus, and cancers (Kristensen et al., 2019). In cashmere goats, a large number of circRNAs have also been identified in skin or hair follicle tissues (Zheng et al., 2020; Shang et al., 2021). Furthermore, circRNA-1926 was revealed to contribute to the differentiation of SHF-SCs into HF cells in cashmere goats through the miR-148a/b-3p/CDK19 axis (Yin et al., 2020). In our recent study, a new circRNA, named circRNA-0100, was identified from the SHFs of cashmere goats, and it was transcribed from the CHD9 gene of goats with significantly higher expression at anagen SHFs than that at telogen (Yin et al., 2019). In fact, CHD9 is a member of the CHD family that is comprised of nine members including CDHs 1–9 (Liu et al., 2021). Although the functional role of CHD9 in SHF-SC fate still needs to be clarified in cashmere goats, it was demonstrated that CHDs play important roles in regulating the differentiation of various stem cells, including embryonic stem cells (Bulut-Karslioglu et al., 2018), neural stem cells (Feng et al., 2017), and mesenchymal stem cells (Mohd-Sarip et al., 2017). To this end, we speculated that circRNA-0100, as a circular RNA molecule transcribed from the CHD9 gene, might be involved in the differentiation of SHF-SCs into hair follicle lineage through certain mechanisms in cashmere goats.

In the present work, we firstly investigated the expression of circRNA-0100 in the SHF bulge of cashmere goats during both anagen and telogen phases. Further, we assessed the potential effects of circRNA-0100 on the differentiation of SHF-SCs into hair lineages in cashmere goats and its possible molecular regulatory mechanism through an RNA pull-down assay along with dual-luciferase reporter assays. Our result from this study will provide novel scientific evidence revealing the potential molecular regulatory mechanisms in the differentiation of SHF-SCs into hair lineage in cashmere goats.

## 2 Materials and methods

### 2.1 Sequence analysis, total RNA of SHF bulge, and cell culture

All experiment protocols were reviewed and approved by the Animal Experimental Committee of Shenyang Agricultural University (Shenyang, China) with ethical code 201606005, and the experiments were conducted following the approved protocol guidelines. The sequence of circRNA-0100 was displayed by BioEdit software (Hall, 1999), within which the potential miRNA targets were analyzed based on three programs (miRDB, RNAhybrid, and miRNA-target) by taking an intersection. We retrieved the corresponding goat miRNA sequence from the miRNA database miRNAsong (https://www2.med.muni.cz/histology/miRNAsong/index.php, last access: 16 April 2021). The total RNA used was isolated from the SHF bulge of cashmere goats in our previous study (Yin et al., 2020). The SHF stem cells were induced for differentiating into hair lineages through co-culturing with dermal papilla cells (DPCs) in a transwell device as described in a previous publication by Yan and colleagues (Yan et al., 2019). Briefly, we firstly seeded the SHF stem cells (passage 3) of cashmere goats on six-well plates, in which we added a transwell insert seeded with DPCs (passage 3) of cashmere goats. And then, under a humidified atmosphere at 37 ${}^{\circ }$ with 5 % CO ${}_{2}$ , the cells were subjected to non-contacting co-culture in fresh DMEM/F12 medium (Hyclone, Logan, UT, USA) that was supplemented with fetal bovine serum (10 %); the culture media was replaced every 2 d (He et al., 2016).

### 2.2 Overexpression of circRNA-0100 and its siRNA interference in SHF-SCs of cashmere goats

The pcDNA3.1 ( + ) circRNA mini-vector (Addgene, Cambridge, MA, USA) was used for overexpression of circRNA-0100 in SHF-SCs, which was carried out as described in our previous study (Yin et al., 2020). Briefly, when the confluence of SHF-SCs reached 80 %, the cells were transfected transiently using the recombinant pcDNA3.1 ( + ) circRNA-0100, which was performed by the Lipofectamine 3000 (Invitrogen, Carlsbad, CA, USA). Simultaneously, other SHF-SCs were subjected to transient transfection with the pMAX-GFP vectors (Addgene, Cambridge, MA, USA) as a negative control group, while a blank control cell group was also set for which the SHF-SCs were not subjected to any treatment. For siRNA interference analysis to circRNA-0100, we designed three specific siRNAs: si-circR-1:5 ${}^{\prime }$ -GAAGUGUCAGAAUCUGCCGCUG-3 ${}^{\prime }$ , si-circR-2:5 ${}^{\prime }$ -AUGAAGUGUCAGAAUCUGCCGC-3 ${}^{\prime }$ , and si-circR-3:5 ${}^{\prime }$ -GUCAGAAUCUGCCGCUGUAAAC-3 ${}^{\prime }$ . These designed siRNAs were commercially synthesized by GenePharma Co., Ltd. (Shanghai, China). Using the siRNAs Lipofectamine RNAiMAX kits (Invitrogen, Shanghai, China), the siRNAs were respectively transfected into SHF-SCs of cashmere goats.

### 2.3 RNA pull-down analysis

Here, we carried out the RNA-pull down analysis according to the method described in published literature (He et al., 2020). Briefly, based on the sequence of black-splice junction site of circRNA-0100, we designed the biotinylated RNA probe with the sequence 5 ${}^{\prime }$ -CAUGAAGUGUCAGAAUCUGCCGCUGUAAACA-3 ${}^{\prime }$ . This RNA probe was chemically synthesized by Sangon Biotech Co., Ltd. (Sangon, Shanghai, China). The analyzed SHF-SCs were harvested and subjected to lysis and sonication. The cellular extract was prepared by cell lysis buffer. Subsequently, the cross-linking was performed through an incubation with the RNA probe at 37 ${}^{\circ }$ C for 8 h. After pre-cleaning, C1 magnetic beads (Life Technologies, Grand Island, NY, USA) were added to the lysates and were subjected to incubating for 1 h at 37 ${}^{\circ }$ C for generating the complex of circRNA-0100/probe/beads. Ultimately, the RNAs were eluted and extracted by the RNAiso reagent kit (TaKaRa, Dalian, China) from which the potential binding miRNAs were detected using the RT-qPCR technique.

### 2.4 Total RNA isolation and primer design

Here, RNAiso kits (TaKaRa, Dalian, China) were utilized for extracting the total RNA from SHF bulges of cashmere goats and its stem cells. For the expression detection of circRNA-0100 and related gene mRNAs, random primers were utilized to synthesize the first strand cDNA with an M-MuLV cDNA synthesis kit (Sangon, Shanghai, China), whereas for the expression detection of miRNAs, the first strand cDNA was synthesized by a One-Step PrimeScript microRNA cDNA synthesis kit (TaKaRa, Dalian, China). The divergent primers for detecting the expression of circRNA-0100 were designed using the CircPrimer program (Zhong et al., 2018). The convergent primers for mRNA detection were designed by the Premier Primer 5.0 program (Premier Biosoft International, Palo Alto, CA, USA). A combined internal control consisting of UBC, YWHAZ, and SDHA was utilized for normalizing the gene expression level (Bai et al., 2014). The corresponding mature sequences of all detected miRNAs were retrieved from the miRNAsong database (https://www2.med.muni.cz/histology/miRNAsong/index.php, last access: 16 April 2021). A combined internal control consisting of let-7d-5p, miR-26a-5p, and miR-15a-5p was utilized for normalizing the miRNA expression level (Bai et al., 2016). All primers are provided in Table 1 with the corresponding detailed information.

**Table 1:** Detail of PCR primers utilized in this study along with the corresponding annealing temperature for PCR amplification.

Genes	Reference	Primer pair with sequence (5 ${}^{\prime }$ –3 ${}^{\prime }$ ) ${}^{a}$	Primer	TA ${}^{b}$	Amplicon
			length (nt)	( ${}^{\circ }$ C)	size ${}^{c}$ (bp)
circRNA-0100	Yin et al. (2019)	F:GCTCGGAGTTGGCATTCATC	20	57	144
		R:TCAACTAATCCGTAACAGGTCAA	20		
CHD9	XM_005691972.3 in GenBank	F:ACTTGGGTGCAGAATTTGAAC	21	55	181
		R:AACTGAGAGTGTGGCGATAAC	21		
Keratin 6	Yin et al. (2020)	F:CAGTCGCAGCCTCTACAACCT	21	56	159
		R:CAAATGCCACCTCCATAACCA	21		
Keratin 7	Yin et al. (2020)	F:GAGTTTGTGGTGTTGAAGAA	20	56	194
		R:AAGTCCAGGGAGCGGTTGTT	20		
Keratin 8	Yin et al. (2020)	F:TCCTTCAGCAGCCGCTCCTA	20	58	160
		R:CTGTAATGCCCCCCAAACCT	20		
Keratin 16	Yin et al. (2020)	F:CCTTTGTGGCTAGTGGTATG	20	55	188
		R:CAGTTTCAGGGGTTGCTTAT	20		
Keratin 17	Yin et al. (2020)	F:GGGGAATGGAAACAGAGGAG	20	56	112
		R:GAGGAGAGAAGCCCAAGATG	20		
KLF5	KU041751 in GenBank	F:CCACCTCCATCCTATGCTGC	20	56	134
		R:TCCAAATCGGGGTTACTCCT	20		
UBC	Bai et al. (2014)	F:GCATTGTTGGGTTCCTGTGT	20	52	90
		R:TTTGCATTTTGACCTGTGAG	20		
YWHAZ	Bai et al. (2014)	F:TGTAGGAGCCCGTAGGTCATCT	22	56	102
		R:TTCTCTCTGTATTCTCGAGCCATCT	25		
SDHA	Bai et al. (2014)	F:AGCACTGGAGGAAGCACAC	19	53	105
		R:CACAGTCGGTCTCGTTCAA	19		
KLF5 ${}^{d}$	The present study	F:TTAGTTTAATTTGTTAGAGAGGTTGTG	27	54	262
		R:TACCCCACRACTACTAACACTTA	23		
miR-20b-5p	MIMAT0036056 in miRNAsong	F:CGCAAAGTGCTCACAGTGCAGGTAG ${}^{e}$	25	63	NA
miR-29a-3p	MIMAT0036113 in miRNAsong	F:CGTAGCACCATCTGAAATCGGTT	23	61	NA
miR-153-5p	MIMAT0035983 in miRNAsong	F:CGTTGCATAGTCACAAAAGTGATC	24	60	NA
miR-24-3p	MIMAT0036091 in miRNAsong	F:CGTGGCTCAGTTCAGCAGGAAC	22	60	NA
let-7c-3p	MIMAT0035885 in miRNAsong	F:CGCTGTACAACCTTCTAGCTTTCC	24	60	NA
let-7d-5p	Bai et al. (2016)	F:CGAGAGGTAGTAGGTTGCATAGTT	24	62	NA
miR-26a-5p	Bai et al. (2016)	F:CGTTCAAGTAATCCAGGATAGGCT	24	61	NA
miR-15a-5p	Bai et al. (2016)	F:CGTAGCAGCACATAATGGTTTGTG	24	63	NA

${}^{a}$ F: forward, R: reverse. ${}^{b}$ TA: annealing temperature. ${}^{c}$ NA: not available. ${}^{d}$ KLF5 was used for the designing of BSP primers. ${}^{e}$ miRNAsong: https://www2.med.muni.cz/histology/miRNAsong/index.php last access: 16 April 2021.

### 2.5 Methylation analysis of the KLF5 gene in SHF-SCs

The gene sequence of goat KLF5 (NC_030819.1, complement: 38595264-38615237) was obtained from goat genomics database (assembly ARS1, https://www.ncbi.nlm.nih.gov/genome/?term=goat, last access: 11 February 2021). And then, potential CpG islands were searched within the 500 nt region of the KLF5 gene prompter by the Methyl Primer Express software (version 1.0, Applied Biosystems, CA, USA). The AliBaba 2.1 software (http://gene-regulation.com/pub/programs.html, last access: 6 March 2021) was utilized for analyzing the potential binding sites of transcription factors. The genomic DNA isolated from analyzed cells was subjected to a treatment of MethylCode™ bisulfite conversion kit (Invitrogen, Shanghai, China). Using bisulfite sequencing PCR (BSP) with primer pairs BSP-F and BSP-R, PCR amplification was conducted with the potential amplified region 262 bp long and with 23 CpG sites. Through the use of pMD18-T Vector (TaKaRa, Dalian, China), we cloned the purified PCR production in *Escherichia coli* DH5 α cells. A total of 10 positive clones were sequenced for each analyzed group of SHF-SCs, and the QUMA procedure (Kumaki et al., 2008) was utilized for measuring and displaying the sequenced results.

### 2.6 Dual-luciferase reporter assays

Here, a dual-luciferase reporter analysis was conducted according to the assay described in the published literature (Yu et al., 2018). Briefly, the 3 ${}^{\prime }$ -untranslated region (3 ${}^{\prime }$ -UTR) fragment of cashmere goat KLF5 mRNA was amplified, which contained the potential binding site of miR-153-3p, and was ligated to the pGL3 basic vector (Promega, Madison, WI, USA). Subsequently, the SHF-SCs (passage 3) of cashmere goats were prepared and subjected to transfection with the KLF5 3 ${}^{\prime }$ -UTR fragment reporter vectors using Lipofectamine 2000 (Invitrogen, Carlsbad, CA, USA). After culture for 48 h, the transfected cells were subjected to consecutive detection of luciferase activity by the Dual-Luciferase Reporter Assay System (Promega, Madison, WI, USA). The luciferase activity ratio of Firefly to Renilla was measured to eliminate deviation from transfection efficiencies.

### 2.7 Data statistical analysis

All data are expressed as mean ± SEM (standard error of the mean), and statistical analysis was conducted using the SPSS 17.0 procedure (SPSS Inc., Chicago, IL, USA). The difference between two groups was compared by Student's t test, and the difference among more than two groups was compared by one-way analysis of variance combined with Bonferroni's test. A P value less than 0.05 was defined as a significant difference.

## 3 Results and discussion

### 3.1 Molecular characterization of circRNA-0100 sequence in cashmere goat SHFs

**Figure 1:**
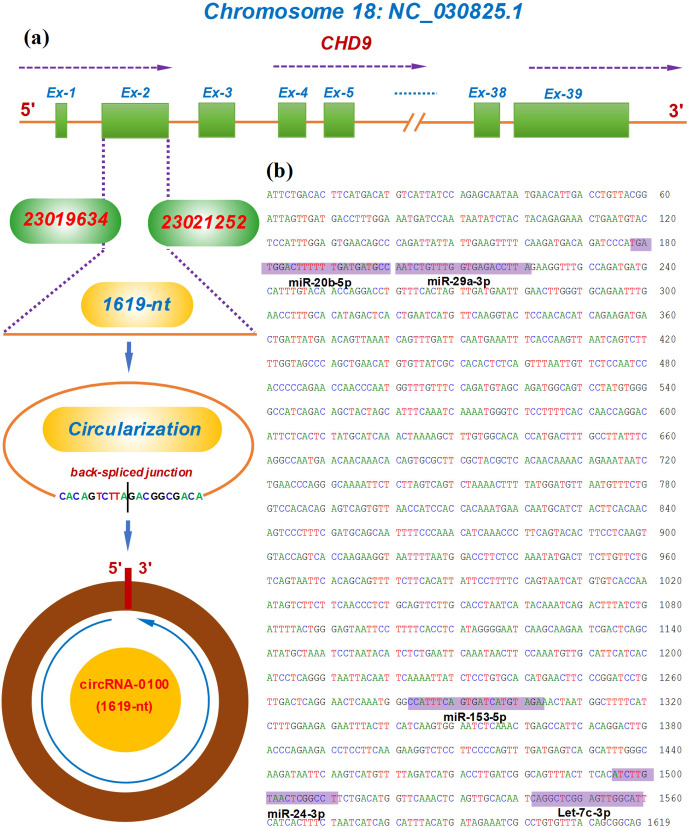
Transcriptional source of circRNA-0100 in cashmere goats and its molecular structural characteristics. **(a)** Overall diagram of the host gene of circRNA-0100 and its reverse splicing pattern with the size 1619 nt. **(b)** Display of a circRNA-0100 cDNA sequence that contains the potential binding sites of five miRNAs indicated by shading with the respective miRNA name.

The circRNA-0100 was previously identified in SHFs of cashmere goats (Yin et al., 2019). Based on the alignment of the circRNA-0100 linearized sequence against the goat genome (https://www.ncbi.nlm.nih.gov/assembly/GCF_001704415.1, last access: 11 February 2021, ARS1), the CHD9 gene was revealed to be the host gene of circRNA-0100 (Fig. 1a). As annotated in the NCBI database (https://www.ncbi.nlm.nih.gov, last access: 18 September 2021), the goat CHD gene contains multiple exons including exon 1 to 39 (Fig. 1a). The circRNA-0100 is formed through the reverse splicing of the entire exon 2 of the goat CHD9 gene with position nos. 23019634–23021252 within the NC_030825.1 sequence on chromosome 18 (Fig. 1a), and it has a spliced length of 1619 nt, which has been verified by PCR amplification with two divergent primer pairs followed by sequencing analysis (Fig. S1). On the other hand, it is thought that circRNAs can exert biological roles through sequestering miRNAs to impede their binding to corresponding target mRNAs (Kristensen et al., 2019). Based on in-silicon analysis, we found that circRNA-0100 contained potential binding sites of five miRNAs, including miR-20b-5p, miR-29a-3p, miR-153-3p, miR-24-3p, and let-7c-3p (Fig. 1b), which suggests that circRNA-0100 may play roles via miRNA-mediated pathways in SHFs of cashmere goats.

### 3.2 Expression characterization of circRNA-0100 in bulge and its effects on the differentiation of SHF-SCs into hair follicle lineages

**Figure 2:**
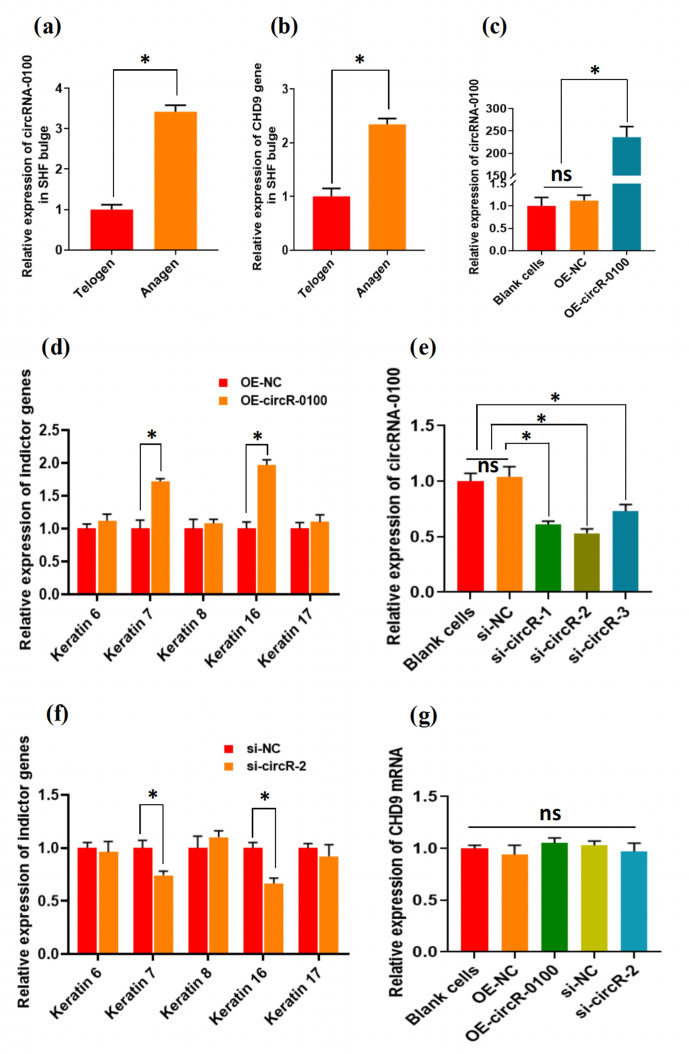
The expression patterns of circRNA-0100 along with its host gene CHD9 in the SHF bugle of cashmere goats and the effects circRNA-0100 on the differentiation of SHF-SCs into hair follicle lineages of cashmere goats. **(a)** Expression pattern of circRNA-0100 in the SHF bugle of cashmere goats. **(b)** Expression pattern of the host gene CHD9 of circRNA-0100 in the SHF bugle of cashmere goats. **(c)** Overexpression efficiency analysis of circRNA-0100 in SHF-SCs of cashmere goats. **(d)** Overexpression of circRNA-0100 caused the significant increase in indictor genes: Keratin 7 and Keratin 16 in SHF-SCs of cashmere goats. **(e)** Knockdown efficiency analysis of si-circR-1, si-circR-2, and si-circR-3 to circRNA-0100 in SHF-SCs of cashmere goats. **(f)** Knockdown of circRNA-0100 caused the significant decrease in indictor genes: Keratin 7 and Keratin 16 in SHF-SCs of cashmere goats. **(g)** Neither overexpression nor knockdown of circRNA-0100 had a significant effect on the expression of its host gene CHD9 in SHF-SCs of cashmere goats.

For characterizing the expression of circRNA-0100 in the bulge of cashmere goat SHF, we selected two main stages: anagen and telogen. As shown in Fig. 2a, circRNA-0100 has significantly higher expression in the anagen bulge in comparison to that of telogen. A highly similar expression pattern was also observed in the expression of its host gene CHD9 (Fig. 2b). It is well known that anagen is a highly active stage, during which the SHF-SCs continuously differentiate into hair follicle lineages under the signal induction from dermal papilla cells in order to drive the SHF regeneration of cashmere goats, which was also supported by the results from our previous study (Yin et al., 2020) Thus, considering the higher expression of circRNA-0100 in the anagen SHF bulge of cashmere goats (Fig. 2a), we speculated that it might play certain roles in regulating the differentiation event of SHF-SCs into hair lineages.

To verify this hypothesis, we conducted gain- and loss-of-function analysis of circRNA-0100 in SHF-SCs of cashmere goats. As observed from Fig. 2c, the overexpression of circRNA-0100 was verified in SHF-SCs for which the relative expression of circRNA-0100 was upregulated approximately 250 times in the overexpression group (OE-circR-0100) compared with the blank cell group (BC) and the negative control group (OE-NC). Subsequently, we tested the expression changes of five indicator genes (including Keratins 6, 7, 8, 16, and 17) in the analyzed cells in order to evaluate the effects of circRNA-0100 on the differentiation process of SHF-SCs into hair lineages. As shown in Fig. 2d, the overexpression of circRNA-0100 led to a significant increase in the expression of both Keratin 7 and Keratin 16. On the other hand, we also conducted a knockdown analysis of circRNA-0100 in SHF-SCs via siRNA interference experiments. Three independent siRNAs were designed and named si-circR-1, si-circR-2, and si-circR-3. Based on the analyzed results of their knockdown efficiency to circRNA-0100 in SHF-SCs (Fig. 2e), si-circR-2 was chosen and used in further knockdown experiments for circRNA-0100. As observed from Fig. 2e, the si-circR-2-mediated knockdown of circRNA-0100 led to a significant decrease in the expression of Keratins 7 and 16 in SHF-SCs compared with their counterparts of the negative control group (si-NC).

Previously, it was recorded that the expression of Keratin 7 is a suitable indictor for the formation of hair follicles at the anagen onset stage (Misago and Narisawa, 2002). Also, it was reported that Keratin 16 initially localizes within early hair germs, suggesting that Keratin 16 indicates cells in a transition state of cellular properties resilient enough to provide the structural integrity required of the early suprabasal layers in the context of hair follicle development (Bernot et al., 2002). Taken together with our results from both overexpression (Fig. 2d) and siRNA interference (Fig. 2e) experiments of circRNA-0100 in SHF-SCs (Fig. 2d and e), it can be inferred that circRNA-1926 may be implicated in contributing to the differentiation of SHF-SCs into hair cells. However, both overexpression and knockdown of circRNA-0100 have no effect on the expression of its host gene: CHD9 in SHF-SCs (Fig. 2g), which suggests that the host gene CHD9 of circRNA-0100 appears not to be implicated in the contributing effects of circRNA-0100 on the differentiation event of SHF-SCs into hair lineages in cashmere goats. Moreover, based on bioinformatics analysis, we could not find any regulatory relationship among circRNA-0100, miRNA, and Keratins 7 and 16.

### 3.3 CircRNA-0100 sequesters miR-153-3p and may negatively regulate its transcript in SHF-SCs of cashmere goats

**Figure 3:**
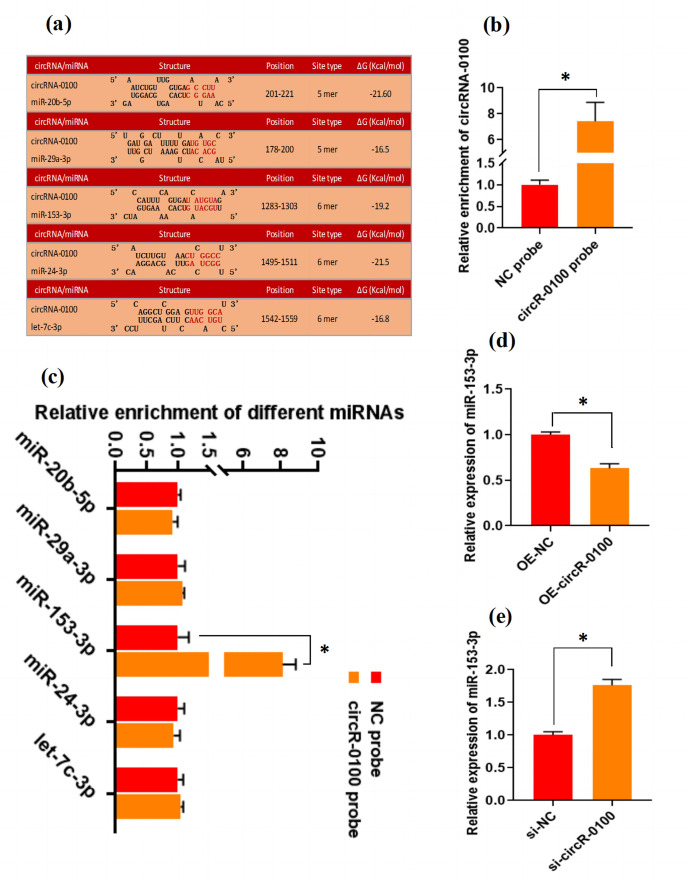
CircRNA-0100 sequesters miR-153-3p and regulates its expression in SHF-SCs of cashmere goats. **(a)** The prediction analysis of the binding sites of miR-20b-5p, miR-29a-3p, miR-153-3p, miR-24-3p, and let-7c-3p within circRNA-0100 sequence. **(b)** Detecting results of circRNA-0100 in the pulled-down pellet by the circR-0100 probe and NC probe. **(c)** Detecting results in the pulled-down pellet by the circR-0100 probe and NC probe. **(d)** Detecting results of miR-153-3p in SHF-SCs subjected to treatment with the circRNA-0100 overexpression assay. **(e)** Detecting results of miR-153-3p in SHF-SCs subjected to treatment with the si-circR-0100 assay. NC probe: biotinylated negative control probe group, circRNA-0100 probe: biotinylated circRNA-0100 probe group, OE-NC: negative control group, OE-circR-0100: overexpression circRNA-0100 groups, si-NC: negative control group, and si-circR-0100: knockdown group of circRNA-0100. The error bar represents the standard deviation within the group. ${}^{*}$ represents significant difference ( P<0.05 ).

There is evidence that circRNAs can sequester natural miRNAs to prevent them from binding with target mRNAs via acting as an miRNA molecular sponge (Hansen et al., 2013; Guo et al., 2014; Yin et al., 2020). In this study, we found that circRNA-0100 harbored the potential binding sites of five miRNAs, including miR-20b-5p, miR-29a-3p, miR-153-3p, miR-24-3p, and let-7c-3p, which was predicted via bioinformatics analysis (Fig. 3a). Subsequently, a biotin-labeled RNA pull-down analysis was carried out to define which miRNAs were sequestered by circRNA-0100. As shown in Fig. 3b, we firstly verified the significantly higher enrichment of circRNA-0100 in the circR-0100 probe pulled-down pellet compared with the counterpart negative control probe (NC probe). Compared with the counterpart of the NC probe, interestingly, a significantly higher enrichment of miR-153-3p was revealed in the circR-0100 probe pulled-down pellet, but not for miR-20b-5p, miR-29a-3p, miR-24-3p, and let-7c-3p (Fig. 3c). It appears apparent that circRNA-0100 sequesters miR-153-3p in SHF-SCs of cashmere goats via serving as its molecular sponge.

Also, we found that the overexpression of circRNA-0100 led to a significant decrease in miR-153-3p in SHF-SCs (Fig. 3d), whereas the knockdown of circRNA-0100 led to a significant increase in miR-153-3p in SHF-SCs (Fig. 3e). However, either the overexpression or knockdown of miR-153-3p did not lead to a significant change in the expression of circRNA-0100 in SHF-SCs (data not shown). These results suggest that circRNA-0100 may regulate the transcription of miR-153-3p in SHF-SCs, although we did not perform an expression correlation analysis between circRNA-0100 and miR-153-3p in SHF-SCs. A highly similar mode of regulation was also reported in research on invasion and metastasis of colorectal cancer; circLONP2 was found to sequester miR-17 and regulate its expression to enhance the invasiveness of colorectal carcinoma cells (Han et al., 2020). Also, in a recent investigation, we found that circRNA-1926 directly combined with miR-148a/b-3p regulated their expression (Yin et al., 2020).

From a functional point of view, to date, it was thought that circRNA may play roles through multiple pathways and mechanisms, such as acting as molecular sponges of miRNAs (Hansen et al., 2013), RNA transport (Ashwal-Fluss et al., 2014), and protein translation (Huang and Shan, 2015). Here, we demonstrated that circRNA-0100 directly combined with miR-153-3p (Fig. 3c) negatively regulated its expression in SHF-SCs of cashmere goats (Fig. 3d and e). However, the resting biological significance of circRNA-0100 in SHF-SCs of cashmere goats needs to be further investigated, such as its potential roles in pluripotency and/or stem maintenance, as well as activation and proliferation of SHF-SCs of cashmere goats.

### 3.4 CircRNA-0100 upregulates the expression of KLF5 but not via modifying the methylation degree of its prompter

**Figure 4:**
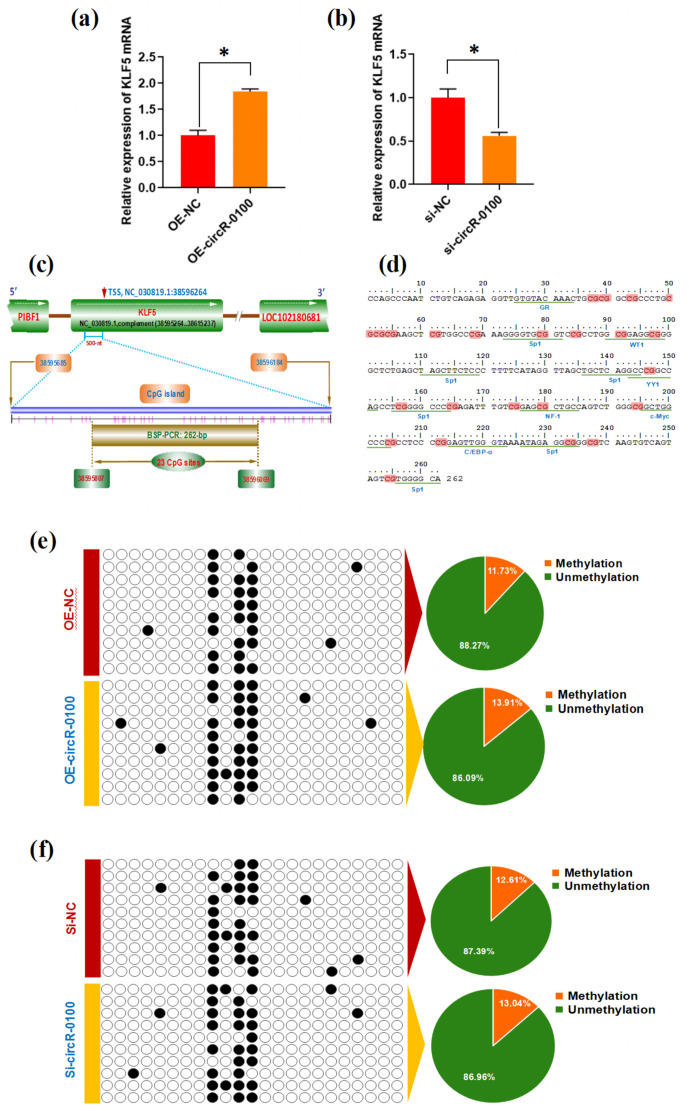
Effects of circRNA-0100 on KLF5 expression and the methylation degree of its promoter in SHF-SCs. **(a)** Detecting results of KLF5 mRNA in SHF-SCs subjected to treatment with the circRNA-0100 overexpression assay. **(b)** Detecting results of KLF5 mRNA in SHF-SCs subjected to treatment with the circRNA-0100 knockdown assay. ${}^{*}$ represents a significant difference ( P<0.05 ). **(c)** A diagram of CpG islands in the KLF5 gene promoter region. The nucleotide positions on the Wnt3a gene are indicated by the NC_030819.1 sequence of the goat genome at NCBI (https://www.ncbi.nlm.nih.gov/genome/?term=goat, last access: 17 April 2021). The CpG sites are represented by short pink vertical lines. **(d)** Potential binding sites of corresponding transcription factors were underlined within the BSP amplification fragment of the KLF5 promoter of goats, and the CpG sites are indicated in shading with dark red. **(e)** Methylation detecting results of the KLF5 promoter in SHF-SCs subjected to treatment with the circRNA-0100 overexpression assay. **(f)** Methylation detecting results of SHF-SCs subjected to treatment with the circRNA-0100 knockdown assay. A total of 23 CpG sites were detected in an amplified fragment 262 bp in length, with 10 clones sequenced for each group. The obtained results are provided in a line for each clone. The filled black circles indicate the methylated CpG sites, and the unfilled white circles indicate the unmethylated CpG sites. Methylation percentage of different SHF-SCs groups is indicated by pie charts. OE-NC: negative control group, OE-circR-0100: overexpression circRNA-0100 groups, si-NC: negative control group, and si-circR-0100: knockdown group of circRNA-0100.

In order to define the regulatory mechanisms of circRNA-0100 in contributing to the differentiation of SHF-SCs into hair lineages, we evaluated the effects of circRNA-0100 on the expression of potential target genes of miR-153-3p in SHF-SCs of cashmere goats. Here, the expression changes of six potential target genes of miR-153-3p were detected in SHF-SCs with overexpression or knockdown of circRNA-0100 including UNC5C, KCNQ4, SERTAD2, KLF5, HEY2, and FEM1C. However, we found that only the KLF5 expression was significantly upregulated (Fig. 4a) or downregulated (Fig. 4b) in SHF-SCs with overexpression or knockdown of circRNA-0100, respectively, but not for the expression of UNC5C, KCNQ4, SERTAD2, HEY2, and FEM1C (data not shown). These results suggest that circRNA-0100 may positively regulate the expression of the KLF5 gene in SHF-SCs through certain unknown mechanisms.

It is widely accepted that DNA methylation within a gene promoter region plays an essential role in regulating its mRNA expression abundance (Daugela et al., 2012). For this reason, we ask whether circRNA-0100 modifies the methylation level of the KLF5 gene promoter, thereby regulating its mRNA expression in SHF-SCs. This promotes us to further investigate the effect of circRNA-0100 on the promoter methylation level of the KLF5 gene in SHF-SCs via overexpression or knockdown techniques on circRNA-0100. Based on bioinformatics screening, a CpG island 500 nt long was revealed immediately upstream of a transcription starting site (TSS) in the goat KLF5 gene (Fig. 4c). Within this CpG island, we amplified a DNA fragment 262 bp long that contained 23 CpG sites and multiple binding sites of potential transcriptional factor (TF), including GR, Sp1, WT1, YY1, NF-1, c-Myc, and C/EBP- α (Fig. 4d). We provide the methylation test results upon the SHF-SCs with overexpression or knockdown of circRNA-0100 in Fig. 4e and f. Unexpectedly, the methylation pattern is highly similar among all analyzed SHF-SCs. Moreover, the ratios of methylated CpG sites are 13.91 % and 11.73 % in the SHF-SCs of overexpression of circRNA-0100 (OE-circR-0100) and negative control (OE-NC) groups, respectively (Fig. 4e). Also, the ratios of methylated CpG sites are 13.04 % and 12.61 % in the SHF-SCs of knockdown of circRNA (Si-circR-0100) and negative control (Si-NC) groups, respectively (Fig. 4f). Taken together with our above results (Fig. 4a and b), it can be suggested that circRNA-0100 upregulates the expression of KLF5 in SHF-SCs but not via modifying the methylation degree of its prompter.

**Figure 5:**
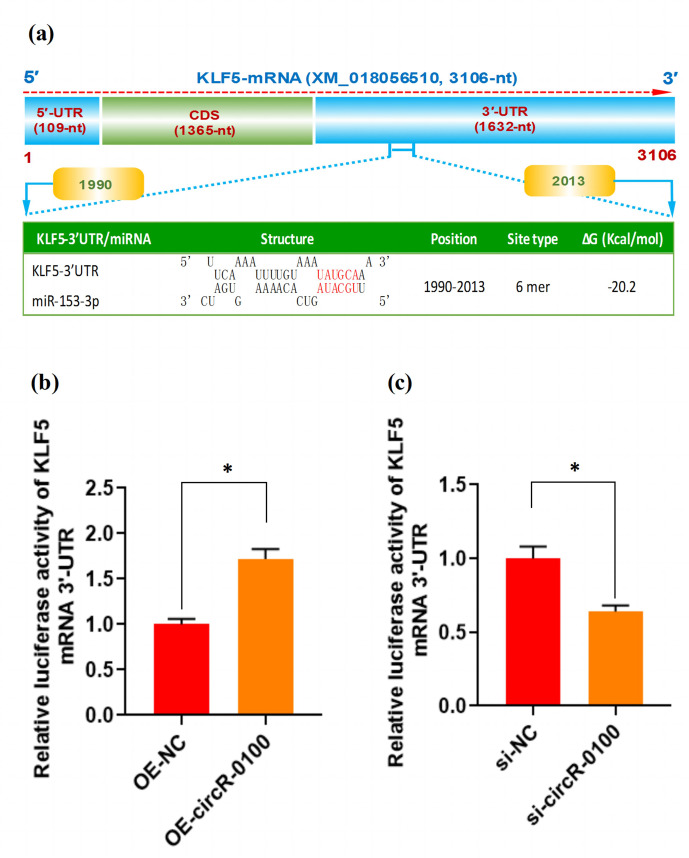
The effect of miR-153-3p on the expression of KLF5 mRNA in SHF-SCs of cashmere goats. **(a)** An overall diagram of goat KLF mRNA and prediction of potential binding sites of miR-153-3p on its mRNA 3 ${}^{\prime }$ -UTR region. The nucleotide positions are indicated based on the goat KLF5 mRNA with accession no. XM_018056510 at NCBI (https://www.ncbi.nlm.nih.gov, last access: 20 April 2021). **(b)** Relative luciferase activities of KLF5 mRNA 3 ${}^{\prime }$ -UTR in SHF-SCs subjected to treatment with the circRNA-0100 overexpression assay. **(c)** Relative luciferase activities of KLF5 mRNA 3 ${}^{\prime }$ -UTR in SHF-SCs subjected to the circRNA-0100 knockdown assay. ${}^{*}$ indicates a significant difference ( P<0.05 ).

**Figure 6:**
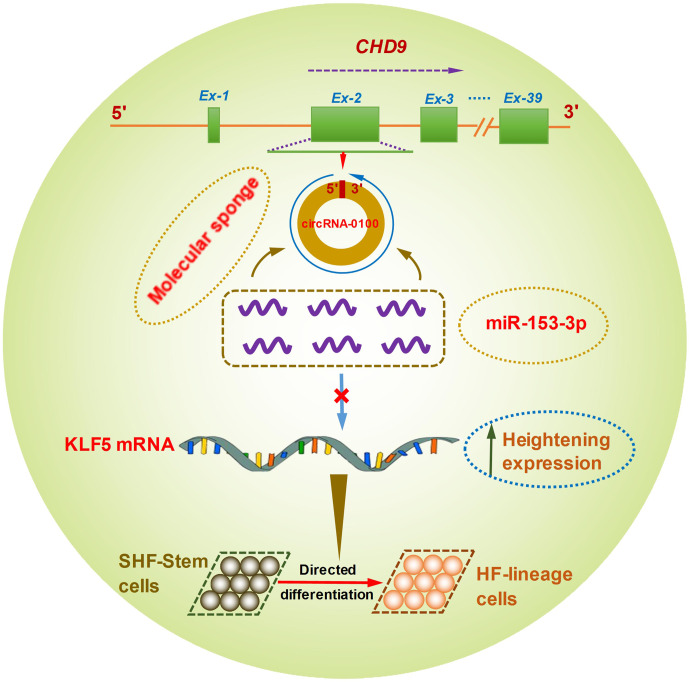
A schematic representation of the functional mechanism of circRNA-0100 in promoting the differentiation of cashmere goat SHF-SCs into hair follicle lineage via sponging miR-153-3p to heighten the KLF5 expression.

However, it was reported that circRNA can regulate the expression of its target gene via altering the methylation level of the promoter region of the same gene (Wang et al., 2018; Liu et al., 2019). An outstanding example exists in human peripheral blood mononuclear cells, wherein circRNA-5692 is found to cause the increasing expression of the DAB2IP gene via reducing the level of its methylation modification in the promoter region (Liu et al., 2019). This revealed function mechanism on circRNAs promoted us to investigate the effect of circRNA-0100 on the promoter methylation level of the KLF5 gene in SHF-SCs. Nevertheless, it is worth noting that, here, we conducted the experiment in SHF-SCs in vitro. Therefore, the results we obtained above should be further confirmed in SHF-SCs in vivo.

### 3.5 CircRNA-0100 upregulates the expression of KLF5 gene via miR-153-3p-mediated pathway

As is well known, circRNA can sponge miRNAs, which causes a reduction of active miRNAs, thereby disinhibiting their target mRNA at the post-transcriptional level (Liu et al., 2018; Yang et al., 2019; Yin et al., 2020). Based on our above results, we confirmed that circRNA-0100 upregulates the expression of KLF5 in SHF-SCs, but not via modifying the methylation degree of its prompter (Fig. 4e and f); moreover, it sequesters miR-153-3p (Fig. 3c). These results indicate a most likely mechanism through which circRNA-0100 may upregulate the expression of the KLF5 gene in SHF-SCs through the miR-153-3p-mediated pathway. Thus, we conducted a bioinformatics screening for potential binding sites of miR-153-3p within 3 ${}^{\prime }$ -UTR of goat KLF mRNA. As shown in Fig. 5a, interestingly, a potential binding site of miR-153-3p was predicted within the 3 ${}^{\prime }$ -UTR region of KLF5 mRNA with a binding type of 6 mer in its seed region. To further confirm this specific binding of miR-153-3p with the 3 ${}^{\prime }$ -UTR region of KLF5 mRNA, we performed a dual-luciferase reporter assay in SHF-SCs of cashmere goats. A 3 ${}^{\prime }$ -UTR fragment of goat KLF5 mRNA was ligated to the reporter vector of dual-luciferase reporter assay, which had the potential binding site of miR-153-3p.

As a result, the overexpression of circRNA-0100 (OE-circR-0100) caused a significant increase in luciferase activity of the 3 ${}^{\prime }$ -UTR fragment of KLF5 mRNA in SHF-SCs in comparison with the negative control (OE-NC) (Fig. 5b), whereas the knockdown of circRNA-0100 (si-circR-0100) caused a significant decrease in luciferase activity of the 3 ${}^{\prime }$ -UTR fragment of KLF5 mRNA in SHF-SCs in comparison with the negative control (si-NC) (Fig. 5c). Taken together with our above results, it can be suggested that circRNA-0100 positively regulated the expression of the KLF5 gene in SHF-SCs through sequestering miR-153-3p.

Although it is not yet known whether KLF5 plays a direct functional role in the differentiation of hair follicle stem cells into hair follicle lineage, it was demonstrated that the expression of KLF5 is enriched at high levels in primary keratinocytes, especially enriched in the cells of the hair matrix and the inner root sheath cuticle layer of the hair follicle (Ohnishi et al., 2000; Sur et al., 2002). Moreover, KLF5 is implicated in the activation of Wnt / β -catenin signaling (Zhang et al., 2020). Interestingly, it was confirmed that the Wnt / β -catenin signaling was deeply implicated in the differentiation of hair follicle stem cells into hair follicle lineages (Choi et al., 2013; Lien et al., 2014; Si et al., 2018). Thus, taken together with above results, we inferred that circRNA-0100 promotes the differentiation of cashmere goat SHF-SCs into hair follicle lineage via sequestering miR-153-3p to heighten the KLF5 expression in SHF-SCs of cashmere goats (Fig. 6).

## 4 Conclusions

For the first time, we confirmed that circRNA-0100 exhibits significantly higher expression in the SHF bulge at anagen in comparison to that at telogen of cashmere goats. Further, we showed that circRNA-0100 might contribute to the differentiation of SHF-SCs into hair follicle lineage in cashmere goats via sequestering miR-153-3p to heighten the KLF5 expression.

## Supplement

10.5194/aab-65-55-2022-supplement The supplement related to this article is available online at: https://doi.org/10.5194/aab-65-55-2022-supplement.

## Data Availability

The datasets analyzed and used in this study are available from the corresponding author upon reasonable request.
