# Supplementary material for: CircRNA-0100 positively regulates the differentiation of cashmere goat SHF-SCs into hair follicle lineage via sequestering miR-153-3p to heighten the KLF5 expression
Source: Arch Anim Breed. 2022 Feb 22;65(1):55–67. doi: 10.5194/aab-65-55-2022 (PMC8889309; doi:10.5194/aab-65-55-2022)
Supplement: The supplement related to this article is available online at: https://doi.org/10.5194/aab-65-55-2022-supplement. [file aab-65-55-supplement.pdf]

Supplement of Arch. Anim. Breed., 65, 55–67, 2022
https://doi.org/10.5194/aab-65-55-2022-supplement
© Author(s) 2022. CC BY 4.0 License.

Open Access
Archives Animal Breeding

Supplement of

CircRNA-0100 positively regulates the differentiation of cashmere goat SHF-SCs into hair follicle lineage via sequestering miR-153-3p to heighten the KLF5 expression

Junyin Zhao et al.

Correspondence to: Wenlin Bai (baiwenlin@syau.edu.cn)

The copyright of individual parts of the supplement might differ from the article licence.

(A)

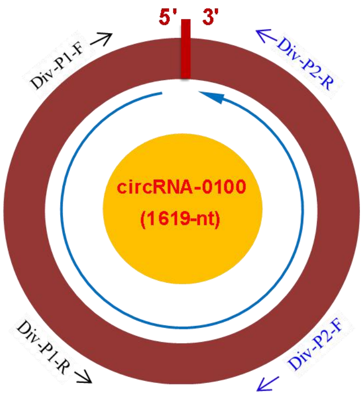

(B)

Div-P1-F: CAGTTGCACAATCAGGCTCG

Div-P1-R: TGTACTGAAGGGTTTGATGT

Div-P2-F: CTGGTCCACACAGAGTCAGT

Div-P2-R: CTGGGCTGTTCACTCCAA

(C)

Div-P1-F vs Div-P1-R, amplicon size:979-bp, *Ta* = 54°C

CAGTTGCACAATCAGGCTCGGAGTTGGCATTCATCACTTTCTAATCATCAGCATTTACATGATAGAAATCGCCTGTGTTTACAGCGGCA[GA]TTCT
GACACTTCATGACATGTCATTATCCAGAGCAATAATGAACATTGACCTGTTACGGATTAGTTGATGACCTTTGGAAATGATCCAATAATATCTACTA
CAGAGAAACTGAATGTACTCCATTTGGAGTGAACAGCCCAGATTATTATTGAAGTTTTCAAGATGACAGATCCCATGATGGACTTTTTTGATGATG
CCAATCTGTTTGGTGAGACCTTAGAAGGTTTGCCAGATGATGCATTTGTACAACCAGGACCTGTTTCACTAGTTGATGAATTGAACTTGGGTGCA
GAATTTGAACCTTTGCACATAGACTCACTGAATCATGTTCAAGGTACTCCAACACATCAGAAGATGACTGATTATGAACAGTTAAATCAGTTTGAT
TCAATGAAATTTCACCAAGTTAATCAGTCTTTTGGTAGCCCAGCTGAACATGTGTTATCGCCACACTCTCAGTTTAATTGTTCTCCAATCCACCCCC
AGAACCAACCCAATGGTTTGTTTCCAGATGTAGCAGATGGCAGTCCTATGTGGGGCCATCAGACAGCTACTAGCATTTCAAATCAAAATGGGTCT
CCTTTTCACCAACCAGGACATTCTCACTCTATGCATCAAACTAAAAGCTTTGTGGCACACCATGACTTTGCCTTATTTCAGGCCAATGAACAACA
AACACAGTGCGCTTCGCTACGCTCACAACAAAACAGAAATAATCTGAACCCAGGGCAAAATTCTCTTAGTCAGTCTAAAACTTTTATGGATGTTA
ATGTTTCTGGTCCACACAGAGTCAGTGTTAACCATCCACCACAAATGAACAATGCATCTACTTCACAACAGTCCCTTTCGATGCAGCAATTTTCCC
AAACATCAAACCCTTCAGTACA

(D)

Div-P2-F vs Div-P2-R, amplicon size: 985-bp, *Ta* = 54°C

CTGGTCCACACAGAGTCAGTGTTAACCATCCACCACAAATGAACAATGCATCTACTTCACAACAGTCCCTTTCGATGCAGCAATTTTCCCAAACA
TCAAACCCTTCAGTACACTTCCTCAAGTGTACCAGTCACCAAGAAGGTAATTTTAATGGACCTTCTCCAAATATGACTTCTTGTTCTGTCAGTAATT
CACAGCAGTTTTCTTCACATTATTCCTTTTCCAGTAATCATGTGTCACCAAATAGTCTTCTTCAACCCTCTGCAGTTCTTGCACCTAATCATACAAAT
CAGACTTTATCTGATTTTACTGGGAGTAATTCCTTTTCACCTCATAGGGGAATCAAGCAAGAATCGACTCAGCATATGCTAAATCCTAATACATCTC
TGAATTCAAATAACTTCCAAATGTTGCATTCATCACATCCTCAGGGTAATTACAATTCAAAATTATCTCCTGTGCACATGAACTTCCCGGATCCTGT
TGACTCAGGAACTCAAATGGGCCATTTCAGTGATCATGTAGAAACTAATGGCTTTTCATCTTTGGAAGAGAATTTACTTCATCAAGTGGAATCTCA
AACTGAGCCATTCACAGGACTTGACCCAGAAGACCTCCTTCAAGAAGGTCTCCTTCCCCAGTTTGATGAGTCAGCATTTGGGCAAGATAATTCAA
GTCATGTTTTAGATCATGACCTTGATCGGCAGTTTACTTCACATCTTGTAACTCGGCCTTCTGACATGGTTCAAACTCAGTTGCACAATCAGGCTC
GGAGTTGGCATTCATCACTTTCTAATCATCAGCATTTACATGATAGAAATCGCCTGTGTTTACAGCGGCA[GA]TTCTGACACTTCATGACATGTCAT
TATCCAGAGCAATAATGAACATTGACCTGTTACGGATTAGTTGATGACCTTTGGAAATGATCCAATAATATCTACTACAGAGAAACTGAATGTACTC
CATTTGGAGTGAACAGCCCAG

Fig. S1 Validation of circRNA-0100 in SHFs of cashmere goat by two divergent primers along with amplicon sequencing. (A) The designing scheme of two divergent primer pairs for amplifying the entire length of circRNA-0100 in SHFs of cashmere goat. (B) The primer sequence of two divergent primer pairs: Div-P1-F vs Div-P1-R, and Div-P2-F vs Div-P2-R. (C) The sequencing result of amplicon by Div-P1-F versus Div-P1-R with annealing temperature (*Ta*). The bases of primer location were displayed with green. The back spliced junction [GA] was indicated in square brackets with red. (D) The sequencing result of amplicon using Div-P2-F versus Div-P2-R with annealing temperature (*Ta*). The bases of primer location were displayed with green. The back spliced junction [GA] was indicated in square brackets with red. Between (C) and (D), the overlapping areas of two amplicon sequences were indicated by sharing shaded areas with the same color. Div-P1-F = Divergent Primer 1 Forward, Div-P1-R = Divergent Primer 1 Reverse, Div-P2-F = Divergent Primer 2 Forward, and Div-P2-R = Divergent Primer 2 Reverse. *Ta* = annealing temperature.
